# Detection of Anatomical Variations and Pathological Findings of Coronary Arteries Using CT Coronary Angiography (CTCA) in Sudanese Adults in a Tertiary Hospital

**DOI:** 10.7759/cureus.93620

**Published:** 2025-09-30

**Authors:** Mohamed Adam, Mahil Abdalla, Esraa Salim, Ragda Abdallah, Arwa Adam, Najla Mohammed, Mohamed A Abdelrahim, Lama Mohamed, Abrar Adam, Mustafa Elnour Hussein Bahar

**Affiliations:** 1 Diagnostic Radiology, Dr. Sulaiman Al Habib Medical Group, Riyadh, SAU; 2 Emergency Medicine, Hull University Teaching Hospitals NHS Trust, Hull, GBR; 3 Internal Medicine, Faculty of Medicine, University of Khartoum, Khartoum, SDN; 4 Paediatrics, Hull University Teaching Hospitals NHS Trust, Hull, GBR; 5 Obstetrics and Gynaecology, Maternity & Children Hospital, Sakakah, SAU; 6 Vascular Surgery, Norfolk and Norwich University Hospitals NHS Foundation Trust, Norwich, GBR; 7 Anatomical Sciences, St. George's University School of Medicine, St. George's, GRD; 8 Internal Medicine, University of Medical Sciences and Technology, Khartoum, SDN; 9 Medical Laboratory and Blood Bank, Omdurman Military Hospital, Omdurman, SDN; 10 Radiology, Faculty of Medicine, University of Khartoum, Khartoum, SDN

**Keywords:** anatomical variations, calcium scoring, coronary anomalies, coronary artery disease, coronary ct angiography

## Abstract

Background: Ischemic heart disease (IHD) is a leading cause of morbidity and mortality worldwide. Traditional diagnostic methods relied on electrocardiography (ECG) and cardiac enzyme testing, with limited accuracy. With advances in imaging, ECG-gated CT coronary angiography (CTCA) has become a reliable, non-invasive tool for risk assessment and diagnosis of coronary artery disease (CAD).

Methods: A retrospective, cross-sectional study was conducted in the radiology department of Ahmed Gasim Hospital, Sudan. Data were extracted from 95 CTCA reports between January 2020 and June 2022 using a structured 14-item questionnaire. Patient demographics, calcium scores, coronary stenosis/occlusion, congenital anomalies, and anatomical variations were analysed.

Results: The mean patient age was 51 years (range: 20-87), with male patients comprising 56.8%. Most patients (63.2%) had a calcium score of zero. Among positive scores, 17.9% were <100, 13.7% were 100-500, 2.1% were 500-1000, and 3.2% were >1000. The left anterior descending artery (LAD) was the most frequently affected artery (20.0%), followed by the right coronary artery (RCA) (13.7%), and left circumflex artery (LCX) (10.5%); left main coronary artery (LMCA) showed no stenosis. Proximal severe stenosis was the predominant pattern. Congenital anomalies were detected in six patients (6.3%), most commonly myocardial bridging. Anatomical variations were also found in five patients (5.3%), with malignant RCA origin from the left cusp being the most frequent.

Conclusion: CTCA effectively identified calcium scores, stenosis, congenital anomalies, and anatomical variations in Sudanese patients. Findings highlight a predominance of proximal severe stenosis in LAD and RCA, with myocardial bridging as the most frequent congenital anomaly and malignant RCA origin as the most common variation.

## Introduction

Ischemic heart disease (IHD) remains one of the leading causes of morbidity and mortality worldwide. Historically, early risk assessment and diagnosis of coronary artery disease (CAD) were limited by technical and physiological constraints in cardiac imaging, with reliance mainly on electrocardiography (ECG) and cardiac enzyme testing [1].

With advances in imaging, electrocardiogram-gated CT coronary angiography (CTCA) has become a reliable non-invasive tool for accurate diagnosis and risk assessment of CAD [1,2]. Compared with invasive coronary angiography, CTCA provides shorter scan times, high-resolution images, and avoids procedural risks. Its major applications include calcium scoring as a marker of CAD, detection of congenital anomalies, evaluation of anatomical variations (particularly malignant variants), and assessment of stenosis or occlusion [2,3].

Previous international studies have highlighted the diagnostic and prognostic utility of CTCA. The Scottish Computed Tomography of the Heart (SCOT-HEART) trial demonstrated that CTCA clarifies diagnosis, guides targeted interventions, and may reduce the risk of future myocardial infarction [3]. Ghadri et al. reported that CTCA was superior to invasive coronary angiography in detecting congenital anomalies, identifying anomalies in 7.9% of patients compared with 2.1% by invasive angiography [2]. Similarly, Chaosuwannakit’s retrospective study in Thailand found a congenital anomaly prevalence of 3.7% among patients undergoing CTCA [4]. Systematic reviews have also compared CTCA with invasive angiography in terms of diagnostic accuracy, prognostic value, and cost-effectiveness, with evidence supporting its role in selected populations [5-8].

In Sudan, however, limited research has been conducted to evaluate coronary artery pathology and anatomical variations using CTCA. This study was therefore designed to investigate coronary calcium scores, stenotic patterns, congenital anomalies, and anatomical variations in Sudanese adults undergoing CTCA at Ahmed Gasim Hospital.

## Materials and methods

Study design and setting

This was a retrospective, cross-sectional hospital-based study analysing CTCA reports. All examinations were performed in the Diagnostic Radiology Department of Ahmed Gasim Specialized Hospital for Cardiology and Renal Transplantation in Khartoum North, Sudan, which is the main tertiary center for cardiac diseases, cardiac surgery, and renal transplantation in the country.

Study population

Inclusion Criteria

The study included adult male and female patients who underwent CTCA at Ahmed Gasim Hospital between January 2020 and June 2022. Both normal and abnormal CTCA reports were eligible. Patients were considered regardless of whether they were clinically healthy or had risk factors for IHD, such as diabetes, hypertension, or hyperlipidemia. Individuals with a known history of angina or IHD were also included. Only cases performed with strict adherence to the CTCA protocol were analysed. Depending on their baseline heart rate, some patients received beta-blockers as part of the preparation protocol, while others did not.

Exclusion Criteria

Patients were excluded if their CTCA reports contained incomplete demographic data, such as missing age. Pediatric patients were not considered, and any study performed without adherence to the standard CTCA protocol was excluded from the analysis.

Sampling

Of 104 available reports, 95 met the inclusion criteria. A total coverage sampling method was used to maximise the detection of rare clinically significant coronary anomalies and anatomical variations, as the number of patients undergoing CTCA in Sudan is relatively limited.

Patient preparation and imaging protocol

Each patient was clinically evaluated prior to the scan, including review of symptoms, past medical history, and risk factors for IHD such as diabetes, hypertension, and hyperlipidemia. Vital signs were assessed on arrival. Depending on their heart rate, some patients received oral or intravenous beta-blockers to reduce the pulse to below 65 beats per minute, unless contraindications such as bradycardia, hypotension, asthma, or atrioventricular block were present [9]. In addition, sublingual nitroglycerin (400-800 µg) was administered in most cases to achieve coronary vasodilatation. Patients were instructed to avoid caffeine for at least 12 hours and to fast for three to four hours before the procedure. Standard precautions were applied to ensure that patients continued their usual cardiac medications. Electrocardiographic (ECG) gating was used during the scan to minimise motion artefacts [10].

Data collection and analysis

Data were extracted from CTCA reports using a structured 14-item questionnaire that was developed by the research team specifically for this study (see Appendices). This tool was not adapted from any copyrighted source and did not require licensing. The questionnaire recorded demographic characteristics, calcium score, stenosis or occlusion status of each major coronary artery, and the presence of congenital anomalies or anatomical variations. Coronary calcium was assessed using the Agatston scoring system [11], which is a widely applied and freely available method for quantifying coronary calcification. Reports were reviewed for both normal and abnormal findings.

All data were entered and analysed using IBM SPSS Statistics for Windows, version 20 (IBM Corp., Armonk, New York, United States). Descriptive statistics were generated. To minimise bias, incomplete or inconsistent records were excluded, and data were cleaned before analysis.

Ethical considerations

Confidentiality was maintained throughout the process by coding patient records and removing all identifying information. Ethical approval was obtained from the Planning and Training Unit, General Directorate of Therapeutic Medicine, Ministry of Health, Khartoum State, Sudan (approval number: MOH/KS/GDTM/85/C).

## Results

Demographic characteristics

A total of 95 patients were included, with ages ranging from 20 to 87 years (mean: 51 years). The most frequent age groups were 45-50 and 60-65 years. Males represented 56.8% of patients, while females accounted for 43.2% (Table 1).

Coronary calcium score

Most patients (60 cases, 63.2%) had a calcium score of 0. Among those with positive scores, 17 patients (17.9%) were <100, 13 patients (13.7%) were between 100-500, two patients (2.1%) were between 500-1000, and three (3.2%) exceeded 1000. For percentile distribution, most positive scores fell between the 76th and 100th percentile (16%). These findings are summarised in Table 1.

**Table 1:** Demographics and calcium score (N = 95) Calcium scores were calculated using the Agatston method [11].

Variable	Category	Frequency (percentage)
Sex	Male	54 (56.8)
	Female	41 (43.2)
Age groups	45–50 years	Peak
	60–65 years	Peak
Calcium score	0	60 (63.2)
	<100	17 (17.9)
	100–500	13 (13.7)
	500–1000	2 (2.1)
	>1000	3 (3.2)

Coronary artery stenosis and occlusion

The distribution of stenosis across the coronary arteries is shown in Table 2.

**Table 2:** Coronary artery stenosis patterns (N = 95) RCA: right coronary artery; LAD: left anterior descending artery; LCX: left circumflex artery; LMCA: left main coronary artery

Vessel	Normal, n (%)	Stenosis, n (%)	Predominant Pattern
RCA	82 (86.3)	13 (13.7)	Proximal severe
LAD	76 (80.0)	19 (20.0)	Proximal severe
LCX	85 (89.5)	10 (10.5)	Proximal/whole
LMCA	95 (100)	0 (0.0)	–

Congenital coronary anomalies

Congenital anomalies were identified in six patients (6.3%) (Table 3). The most common was myocardial bridging (three cases, 3.2%), followed by congenital absence of left circumflex artery (LCX) with right coronary artery (RCA) takeover (two cases, 2.1%), and absence of RCA with LCX takeover (one case, 1.1%).

Anatomical variations

Anatomical variations were also present in five patients (5.3%) (Table 3). The most frequent was malignant RCA origin from the left aortic cusp passing between the aortic root and pulmonary artery (three cases, 3.2%). Rare variations included LCA arising from the right coronary sinus (one case, 1.1%) and left anterior descending artery (LAD) arising from the right coronary sinus (one case, 1.1%).

**Table 3:** Congenital anomalies and anatomical variations (N = 95) RCA: right coronary artery; LAD: left anterior descending artery; LCX: left circumflex artery

Category	Finding	Frequency (Percentage)
Congenital anomalies	Myocardial bridging	3 (3.2)
	Absence of LCX with RCA takeover	2 (2.1)
	Absence of RCA with LCX takeover	1 (1.1)
Anatomical variations	RCA from left cusp (malignant course)	3 (3.2)
	LCA from the right coronary sinus	1 (1.1)
	LAD from the right coronary sinus	1 (1.1)

## Discussion

This study assessed anatomical variations and pathological findings of coronary arteries in Sudanese adults using CTCA. The results demonstrate that CAD is more prevalent in males, particularly in the age groups 45-50 and 60-65 years, which aligns with global epidemiological patterns [1,3].

Calcium score

The majority of patients (63.2%) had a calcium score of zero, while most positive scores were in the low range (<100; 17.9%). Only 5.3% had scores above 500. This indicates that many Sudanese patients undergoing CTCA for suspected IHD still show relatively low calcium scores. Similar distributions were reported internationally; for instance, the SCOT-HEART trial confirmed that calcium scoring enhances risk stratification, particularly when scores are low to moderate [3].

Coronary artery stenosis

The LAD was the most frequently affected vessel (20% stenosis prevalence), followed by the RCA (13.7%) and LCX (10.5%), while the left main coronary artery (LMCA) showed no stenosis. Proximal severe stenosis was the predominant pattern across vessels. These findings partially differ from the international literature. These findings partially differ from the international literature. For example, a study from Thailand reported RCA involvement as more prominent [4]. The absence of LMCA stenosis in this Sudanese cohort contrasts with reports estimating 6% LMCA involvement worldwide [2,5]. 

The predominance of proximal severe stenosis in this cohort has important clinical implications, since proximal lesions endanger large myocardial territories and carry a higher risk of adverse outcomes. Such high-risk lesions often require more complex PCI techniques, including lesion preparation, debulking, and specialised revascularisation approaches, to achieve optimal results [12,13].

Congenital anomalies

Congenital anomalies were present in six patients (6.3%), with myocardial bridging being the most common (3.2%). This aligns with prior studies reporting myocardial bridging as the leading congenital anomaly, with prevalence rates under 5% in most populations [2]. Rare anomalies such as the absence of LCX or RCA with contralateral takeover were also detected. 

Anatomical variations

Anatomical variations were also observed in five patients (5.3%). The most frequent was malignant RCA origin from the left coronary cusp with an inter-arterial course between the aortic root and pulmonary artery (3.2%). This finding is clinically significant, as such malignant courses have been linked to sudden cardiac death in young patients [2,14]. Internationally, trifurcation of the LAD is often reported as the most common variation, with the incidence ranging from 0.3% to 1.6% [4]; however, this was not observed in the present study.

Comparison to the literature

Overall, the distribution of calcium scores, stenosis patterns, and anomalies in this Sudanese cohort parallels several global findings but also highlights unique trends, including the absence of LMCA stenosis and a relatively high detection rate of malignant RCA variations. These differences may reflect ethnic, genetic, or environmental factors, as well as sample size limitations. The distribution of stenosis in this study also corresponds with the broader pathophysiological understanding of CAD described in standard cardiology references, such as Braunwald’s Heart Disease textbook [15].

Limitations

This study was limited by its retrospective single-centre design and modest sample size. The restricted number of modern CT scanners and trained radiologists in Sudan constrained the availability of CTCA, limiting generalizability. Additionally, only descriptive analysis was performed; larger studies with inferential statistics are needed to better assess associations and predictive factors.

Recommendations

Larger studies are needed to validate these findings across Sudan. Expansion of CTCA services is essential to overcome limitations of access and expertise. Radiology training programs should prioritise CTCA interpretation to address workforce shortages, and wider adoption of CTCA in suspected CAD may improve diagnostic accuracy, risk stratification, and clinical outcomes.

## Conclusions

CTCA in Sudanese adults effectively identified calcium scores, coronary stenosis, congenital anomalies, and anatomical variations. LAD was the most frequently stenosed vessel, predominantly with proximal severe stenosis. Myocardial bridging was the most common congenital anomaly, while malignant RCA origin was the leading anatomical variation.

## Appendices

Study questionnaire

**Table 4:** Research Questionnaire Form

Section	Item	Response Options / Notes
Administrative data	Report form number	
	Date	___ / ___ / ___
Demographics	Age	____ years
	Sex	Male / Female
Calcium Score	Score percentile	0–25 / 26–50 / 51–75 / 76–100
	Agatston score	0 / Mild / Moderate / Obstructive
Coronary Vessels	RCA	Normal / Mild stenosis / Moderate / Severe
	LMCA	Normal / Mild stenosis / Moderate / Severe
	LAD	Normal / Mild stenosis / Moderate / Severe
	LCX	Normal / Mild stenosis / Moderate / Severe
Anomalies	Presence	Yes / No
	Type	Benign / Malignant
Anatomical Variations	Presence	Yes / No
	Type	Benign / Malignant
